# Oxidative stress and repetitive element methylation changes in artisanal gold miners occupationally exposed to mercury

**DOI:** 10.1016/j.heliyon.2017.e00400

**Published:** 2017-09-12

**Authors:** Diana M. Narváez, Helena Groot, Sonia M. Diaz, Ruth Marien Palma, Nathalia Muñoz, Marie-Pierre Cros, Hector Hernández-Vargas

**Affiliations:** a Human Genetics Laboratory. Universidad de los Andes. Bogotá, Colombia; b Instituto Nacional de Salud (INS). Bogotá, Colombia; c Epigenetics Group. International Agency for Research on Cancer (IARC). Lyon, France

**Keywords:** Public health, Toxicology, Genetics, Biological sciences, Health sciences

## Abstract

Mercury (Hg) exposure is a public health concern due to its persistence in the environment and its high toxicity. Such toxicity has been associated with the generation of oxidative stress in occupationally exposed subjects, such as artisanal gold miners. In this study, we characterize occupational exposure to Hg by measuring blood, urine and hair levels, and investigate oxidative stress and DNA methylation associated with gold mining. To do this, samples from 53 miners and 36 controls were assessed. We show higher levels of oxidative stress marker 8-OHdG in the miners. Differences in *LINE1* and *Alu(Yb8)* DNA methylation between gold miners and control group are present in peripheral blood leukocytes. *LINE1* methylation is positively correlated with 8-OHdG levels, while *XRCC1* and *LINE1* methylation are positively correlated with Hg levels. These results suggest an effect of Hg on oxidative stress and DNA methylation in gold miners that may have an impact on miners’ health.

## 1 Introduction

Mercury (Hg) is a heavy metal with a known impact on the environment and on human health. Some of the toxic effects of Hg are caused by increased production of reactive oxygen species (ROS), causing oxidative stress (Park and Zheng, 2012). Oxidative stress is due to dysregulation of the homeostasis between ROS and cell antioxidant and repair mechanisms. It has been shown that Hg exposure inhibits antioxidant enzymes like glutathione-S-transferase (GST), glutathione peroxidase (GPx), superoxide dismutase (SOD) and catalase (CAT) (Martinez et al., 2014; Zeneli et al., 2016) increasing oxidative stress in the cell. Due to its chemical properties, Hg has been used in various applications including artisanal gold mining (Crespo-López et al., 2009) eliciting an occupational exposure that could lead to increased oxidative stress. During the mining process elemental Hg (Hg°) vapour is generated when Hg-gold amalgam is burned to release pure gold (Gibb and O’Leary, 2014). When inhaled, Hg° is oxidized in the red blood cells and lungs to divalent inorganic cation (Hg^+2^), while inhalation of Hg° vapour mainly deposits in the kidney and brain (Park and Zheng, 2012). In the environment, Hg° vapour settles in soil and water near the mining zones. In the sediments, Hg is transformed into methylmercury (MeHg) by the action of anaerobic microorganisms (Parks et al., 2013). MeHg bioaccumulates and biomagnifies in the trophic chain, and in consequence, the general population could be environmentally exposed to MeHg through fish consumption (Li et al., 2010). Therefore, Hg exposure has become a major public health concern in developing countries due to the growing use of this metal, its persistence in the environment, and its high toxicity (Kristensen et al., 2014).

Environmental chemicals, including Hg, can produce abnormal DNA methylation status. These epigenetic changes are induced by oxidative stress or cell metabolism disruption (Fragou et al., 2011). The understanding of these changes is a prerequisite to implement new biomarkers for prevention of diseases related to occupational exposure. Some experimental studies have demonstrated that Hg exposure can change DNA methylation patterns; although, the molecular mechanism involved in Hg epigenetic effect is still unknown (Ruiz-Hernandez et al., 2015). In addition, few human studies have investigated the relationship between environmental Hg exposure and DNA methylation. For instance, Hanna and collaborators have shown an increased methylation in the promoter of *GSTM1* when measured in women with high blood Hg levels (Hanna et al., 2012). Another study correlated DNA hypomethylation of the seleno-protein P plasma 1 (*SEPP1*) promoter, an important enzyme involved in the antioxidant response in the cell, and increasing Hg levels measured in hair (Goodrich et al., 2013). On the other hand, environmental factors can alter methylation of DNA repair genes and modulate base-excision repair (BER) activity increasing oxidative DNA damage susceptibility (Langie et al., 2014). The effects of oxidative stress conditions in methylation have been demonstrated in DNA repair genes, but few studies involved populations exposed to environmental chemicals, and none were associated with Hg exposure. These findings suggest a possible epigenetic effect of Hg in humans, but do not elucidate the effect of Hg on DNA methylation; therefore, further investigation is needed.

DNA methylation is an important epigenetic mechanism involved in gene regulation, and it is often associated with transcriptional silencing of genes. Therefore, research in this field may contribute to support the use of epigenetic marks as possible biomarkers of chemical exposure in epidemiological studies. It has been documented that hypermethylation both in 8-Oxoguanine glycosylase *(OGG1)* and in X-ray repair cross complementing 1 (*XRCC1)* CpG islands may be associated with oxidative stress conditions. More specifically, decrease in *OGG1* mRNA and protein was linked with hypermethylation of the first exon CpG island of this gene in age-related cataract patients, where oxidative stress and ROS play a critical role in pathogenesis (Wang et al., 2015). Similarly, supressed expression of *XRCC1,* and *OGG1* mRNA and proteins was detected during Cadmium-induced malignant transformation of human bronchial cells in association with promoter CpG island hypermethylation (Zhou et al., 2012). This information suggests that epigenetic changes may regulate the DNA repair process under oxidative stress conditions by inactivating repair genes, which may play an important role in Hg-induced toxicity.

In addition to analysing gene-specific DNA methylation, it is important to provide a global methylation status within the genome to better understand the epigenetic effects of environmental exposures. Global methylation changes are often quantitated using repetitive elements. There are about half a million long interspersed nucleotide elements (LINE1), and almost 1.4 million Alu repetitive elements in the human genome (Yang et al., 2004). These repetitive elements are heavily methylated and contribute to more than one-third of DNA methylation; therefore, methylation in repetitive elements may be used as a surrogate marker of global methylation (Yang et al., 2004). Furthermore, systemic changes in global methylation may be measured in blood samples and would be useful as biomarkers of effect in exposed populations.

In light of these observations, the aim of this study was to investigate oxidative stress, and DNA methylation associated with artisanal gold miners as potential biomarkers of occupational exposure to Hg. The exposure was characterized by measuring levels of total Hg in blood, urine and hair samples. Oxidative stress induced by Hg exposure was determined by measuring 8-hydroxy-2' −deoxyguanosine (8-OHdG) levels in genomic DNA from peripheral blood samples. In addition, methylation of the repetitive elements *LINE1* and *AluYb8,* as well as the promoter region of antioxidant genes associated with Hg exposure *SEPP1, SOD2* and DNA repair genes *OGG1*, *XRCC1,* involved in cellular response to oxidative stress, was analysed in DNA from white blood cells.

## 2 Methods

### 2.1 Study population

A cross-sectional study was conducted in a population exposed to Hg in the northern region of Colombia (*La Mojana)*, which consisted of miners dedicated to artisanal gold extraction that have been working for at least 6 months prior to the time the samples were taken (*n* = 53). A control group was included from permanent residents of the region who were not artisanal gold miners (*n* = 36). Since all participants were residents of the same region, environmental Hg exposure was considered the same for both groups. As mining activities often take place near rivers, and frequent fish consumption is characteristic of the diet of local riverine population; therefore, Hg exposure (in its MeHg form) could additionally occur through intake of contaminated fish.

This study was reviewed and approved by the Research Ethics Committee of the Los Andes University (Colombia), minute number 459 2015. All participants have voluntarily accepted to participate and signed an informed consent. A survey was applied to all participants with a questionnaire that included demographic information, as well as fish consumption and smoking history to control the effect of these confounding factors. Smoking may be a confounding factor for oxidative stress by increased ROS production in smoke, and also by weakening the antioxidant defence systems (Dasgupta and Klein, 2014). Blood, urine and hair samples were taken from each participant.

### 2.2 Determination of Hg levels in biological samples

Total Hg levels were measured in blood, hair and urine samples. Blood samples were collected in heparin or EDTA tubes (BD) by venepuncture, and were stored on ice until analysis. Approximately 20 strands of hair 1 cm in length (about 10 mg), were gathered and cut from the occipital region of the scalp. Each sample was placed in plastic bags and maintained at room temperature. Urine samples were collected in 50 mL sterile polyethylene tubes, and were kept at 4 °C. Chemical analyses were carried on using Cold Vapor-Atomic Absorption Spectrophotometry (CV-AAS), using certified reference materials for quality control (IAEA-085, International Atomic Energy Agency, Vienna, Austria; and SRM 3668 and 955c, National Institute of Standards and Technology, Bethesda, MD). Results are given either as μg/L or as μg/g of dry weight.

### 2.3 DNA extraction

Peripheral blood samples were collected in EDTA tubes (BD) by venepuncture. White blood cell layer was isolated by centrifugation for DNA extraction. Genomic DNA was extracted using the FlexiGene DNA kit (QIAGEN Hombrechtikon, Switzerland), adhering to the protocol recommended by the manufacturer.

### 2.4 8-OHdG ELISA assay

Oxidative stress was determined measuring 8-OHdG levels in DNA by an 8-OHdG ELISA kit (Abcam, Boston, MA). Briefly, a DNA aliquot (50 μg) was digested using nuclease P1. Samples’ pH was adjusted with 1 M Tris, and digested with alkaline phosphatase. Digested DNA was then boiled for 10 min and kept on ice until use. Standard and samples were added to the ELISA plate along with previously diluted 8-OHdG-antibody preparation, and incubated for 1 h at room temperature. The plate was washed and the substrate was added. The ELISA plate was developed in the dark for 30 minutes and read on a BioRad micro plate reader at a wavelength of 450 nm.

### 2.5 Bisulfite pyrosequencing

The promoter region of *SEPP1, SOD2, OGG1*, and *XRCC1*, and the repetitive elements *LINE1* and *AluYb8,* were assessed in all samples (Table 1). Genomic DNA (250 ng) was bisulfite modified by the EZ DNA Methylation-Gold Kit (Zymo Research Corporation, Irvine, CA, USA) as per the manufacture’s instructions. Gene regions were amplified using PCR (HotStarTaq Master Mix kit − Qiagen) under the following conditions: initial denaturation at 95 °C for 15 s; 50 cycles of denaturation at 95 °C for 30 s, annealing temperature (See Table 1) for 30 s, and extension at 72 °C for 1 min; final extension at 72 °C for 10 min. Then, the PCR product was sequenced by pyrosequencing using the PyroMark ID Q96 system (Qiagen and Biotage, Uppsala, Sweden). For all genes, percentage DNA methylation at individual CpG sites, as well as average percentage of DNA methylation was analysed.

**Table 1:** Detailed Bisulfite Pyrosequencing Analyses.

Gene	PCR Primer sequence	Annealing T. (°C)	PCR product (bp)	Sequencing Primer	CpG sites
*AluYb8*	F: 5′-AGATTATTTTGGTTAATAAG-3′ R (Bt): 5′-AACTACRAACTACAATAAC-3′	49	178	1: 5′-GTTTGTAGTTTTAGTTATT-3′	5
*LINE1*	F (Bt): 5′-TAGGGAGTGTTAGATAGTGG-3′ R: 5′-AACTCCCTAACCCCTTAC-3′	58	109	1: 5′-CAAATAAAACAATACCTC-3′	7
*OGG1*	F: 5′-GTGGTTTTGAAGAYGGATAGT-3′ R (Bt): 5′-CTCCAAAAACAAACCACAAC-3′	58	267	1: 5′-AGTTTTGAGGAAT-3′ 2: 5′-GAGAGTTTAGTGT-3′	7 8
*SEPP1*	F: 5′-GAAATTGTGTATTYGGGGAGT-3′ R (Bt): 5′-ACTCTAACAAAACATTCCACC-3′	58	307	1: 5′-ATTGATAGATATAGA-3′ 2: 5′-GTTAGTTTGAGTGA-3′	4 5
*SOD2*	F: 5′-GTTAGTGTTGGTGTTATYGTTGATG-3′ R (Bt): 5′-ACTAACCTACAACCTCCTTTCTC −3′	58	293	1: 5′-AGTGTTGGTGTTAT-3′ 2: 5′-AGTTATTATAGTTA-3′ 3: 5′-GGGGAGTAGGGT-3′ 4: 5′-GGGGGTGTTTTG-3′	5 8 10 9
*XRCC1*	F: 5′-GGTTAGAAGGATGAGGTAGAG-3′ R (Bt): 5′-ATCRCTTCTATTACTAAACTCCC-3′	55	309	1: 5′-GTTGGTTAAAGTG-3′ 2: 5′-GATATTGYGTAAGT-3′	13 8

F: Forward primer; R: Reverse primer; Bt: Biotinylated primer.

### 2.6 Statistical analysis

Mean and standard error (SE) were calculated for continuous variables. Unpaired t test or Mann–Whitney U-test for independent samples was performed, according to the results of Shapiro-Wilk normality test, to evaluate differences between control and Hg exposed miners. Two-tailed Spearman–Rho test was used to determine bivariate correlations between methylation status, oxidative stress marker and total mercury levels. *P* values < 0.05 were considered statistically significant. Graphics and analyses were conducted with PRISM version 7.0 (GraphPad Software Inc., San Diego, CA) and STATA version 12 (Stata Corporation, College Station, TX).

## 3 Results

### 3.1 General characteristics

A general description of the population is presented in Table 2. Exposed and control subjects were the same age (approximately 35 years old). The control group consisted of more women (66.67%) than the exposed group (3.77%), which reflects the mainly masculine workforce present in the gold extraction process. Even though there was a clear difference for men and women proportions within the control and exposed groups, when comparing men vs. women no statistical differences were found for the variables studied (Hg, 8-OHdG, and DNA methylation levels, *p* > 0.05). Regarding smoking habits, only the 6.45% of controls and the 11.32% of exposed were smokers. Cumulative smoking habit (package/year) within the smokers was higher for the gold miners, although no significant differences were found when compared to the controls. When participants were asked about fish consumption, the majority of controls (38.89%) report to consume fish 2 to 4 times per week, while most of the miners (47.17%) report a consumption frequency of once per month, being significantly less than the controls.

**Table 2:** Demographic characteristics of the study population.

	Control (*n* = 36)		Gold miners (*n* = 53)		*p* value
	*n* (%)	Mean ± SE	*n* (%)	Mean ± SE	
Age (years)		35.19 ± 2.83		34.38 ± 1.51	0.783
Sex					**<0.001**
Men	12 (33.33)		51 (96.23)		
Women	24 (66.67)		2 (3.77)		
Time of exposure (years)				7.72 ± 1.43	
Smoking habit					0.585
Non smoker	7 (22.58)		15 (28.30)		
Ex-smoker	22 (70.97)		32 (60.38)		
Smoker	2 (6.45)		6 (11.32)		
Cumulative smoking: Package/year		2.90 ± 1.10		4.52 ± 2.37	1.000
Frequency of fish consumption					**0.01**
Never	0		3 (5.66)		
Once per month	7 (19.44)		25 (47.17)		
Once per week	11 (30.56)		14 (26.42)		
2–4 times per week	14 (38.89)		10 (18.87)		
Everyday	4 (11.11)		1 (1.89)		

SE: Standard Error. χ^2^ test for frequencies. Mann–Whitney U-test.

**Fig. 1:**
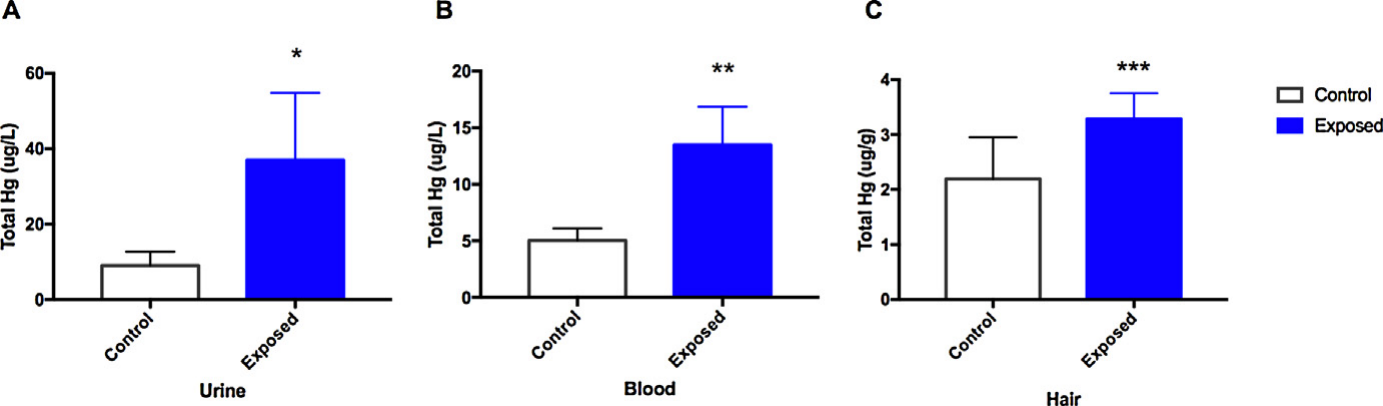
Characterization of Hg exposure. Mean concentration of total Hg levels in the exposed gold miners and the control group. (A) Hg levels in urine, (B) blood, and (C) hair samples. Mann-Whitney U test (*) p < 0.05; (**) p < 0.01; (***) p < 0.001.

Hg exposure was measured as total Hg in blood, urine and hair samples (Fig. 1). Urine Hg values ranged from 3.4 to 785.5 μg/L, while mean Hg levels in urine were significantly higher in the occupationally exposed group (36.98 ± 17.79 μg/L) when compared to the control group (9.02 ± 3.64 μg/L). Hg concentration in blood ranged from 0 to 160.32 μg/L, with a significant higher mean for the exposed group (of 13.46 ± 3.39 μg/L) compared to the control group (5.02 ± 1.08 μg/L). Regarding the Hg hair analyses, total Hg levels range from 0.31 to 24.87 μg/g. Mean Hg levels were again higher for the exposed group (3.28 ± 0.47 μg/g) when compared to the controls (2.19 ± 0.76 μg/g). Hg levels in blood and urine were significantly correlated (Spearman r = 0.436, *p* < 0.01), as well as blood and hair Hg levels (Spearman r = 0.764, *p* < 0.001), and the levels in urine and hair (Spearman r = 0.301, *p* < 0.01).

### 3.2 Oxidative stress in gold miners

**Fig. 2:**
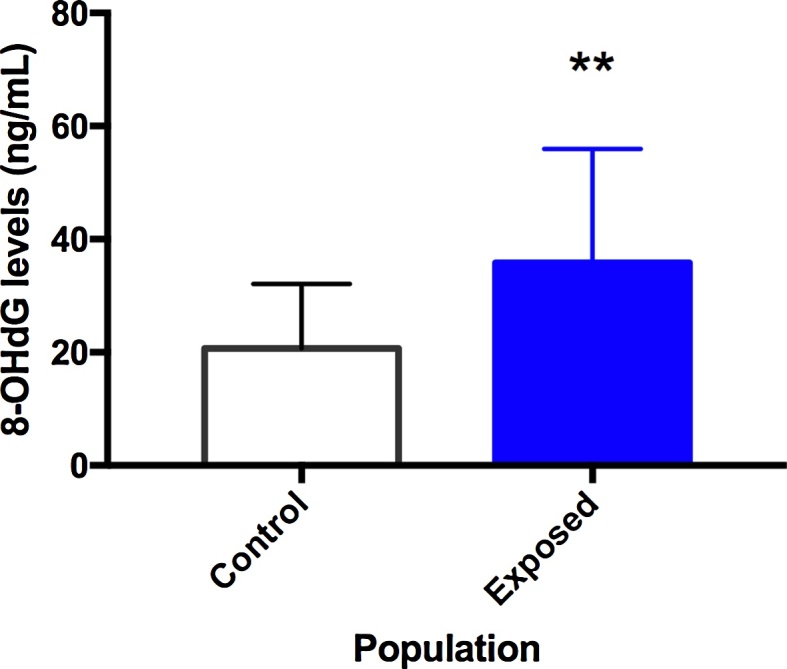
Oxidative stress induced by Hg occupational exposure. Mean oxidative DNA damage (8-OHdG) as biomarker of oxidative stress in peripheral blood samples of Hg exposed gold miners and control group. Mann-Whitney U test (**) p < 0.01.

One of the main effects of Hg exposure is the oxidative stress induction, thus the levels of 8-OHdG in genomic DNA from blood samples were assessed. In this case, occupational exposure to Hg in the gold extraction process led to a statistically significant increase (*p* = 0.003) in the 8-OHdG levels in genomic DNA with a mean of 35.85 ± 3.87 ng/mL when compared to the control group with a mean of 20.68 ± 2.62 ng/mL (Fig. 2).

### 3.3 DNA methylation in repetitive elements

**Fig. 3:**
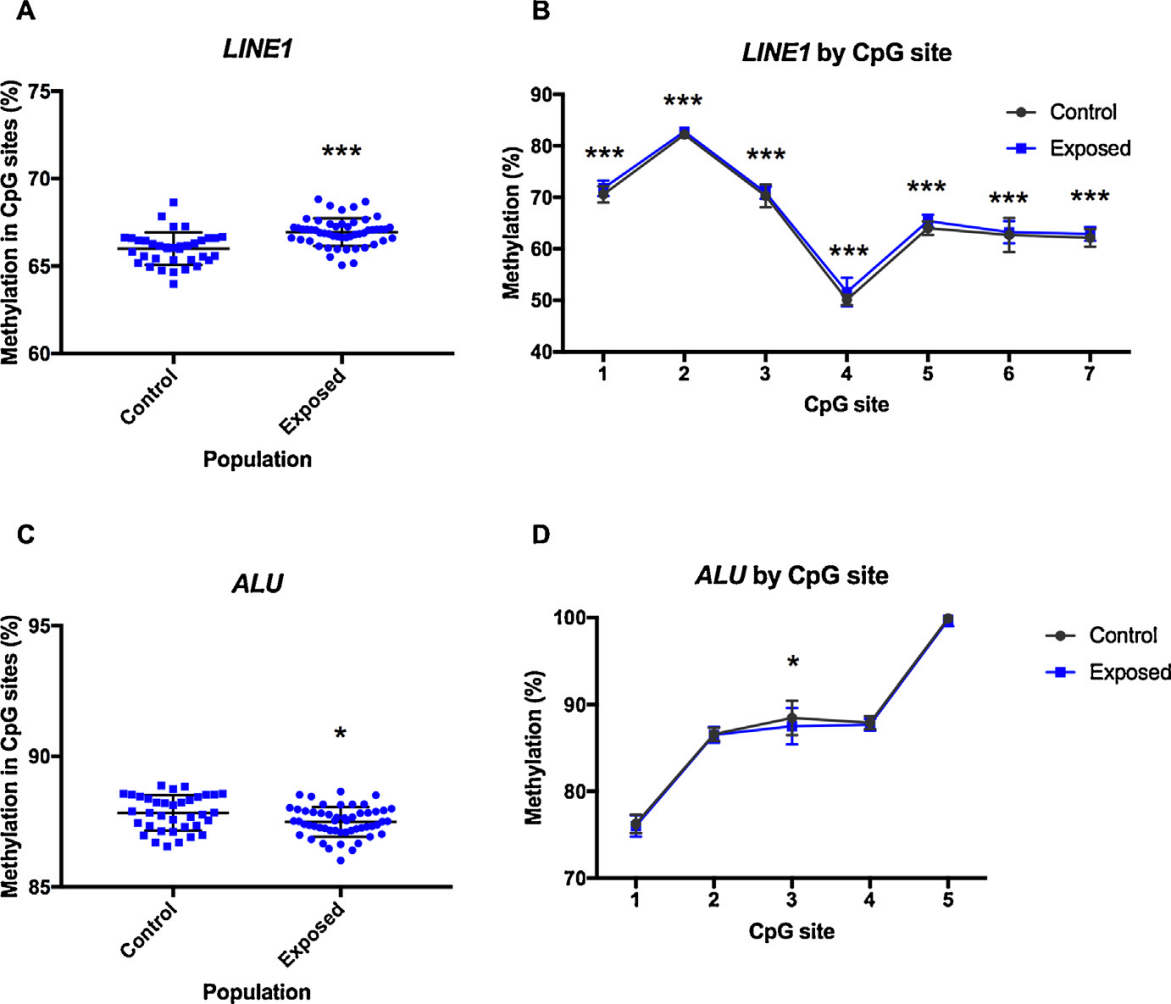
DNA methylation of repetitive elements. DNA methylation in repetitive elements of Hg exposed gold miners and control group assessed by pyrosequencing. (A) Average levels of DNA methylation in *LINE1* from genomic DNA of peripheral blood samples. (B) Levels of DNA methylation in specific CpG sites of *LINE1*. (C) Average levels of DNA methylation in *Alu(Yb8)* from genomic DNA of peripheral blood samples. (D) Levels of DNA methylation in specific CpG sites of *Alu(Yb8)*. Unpaired t test, (*) p < 0.05; (***) p < 0.001.

Global DNA methylation was measured by bisulfite pyrosequencing in *LINE1* and *Alu(Yb8)* repetitive elements (Fig. 3). Average percentage of DNA methylation in *LINE1* was of 65.94 ± 0.16 for the controls and 66.90 ± 0.11 for the miners (Fig. 3A). This significant hypermethylation (*p* < 0.001) in the miners group is consistent for the seven CpG sites analysed for *LINE1* (Fig. 3B). When methylation status was evaluated in *Alu(Yb8),* the average percentage of DNA methylation was of 87.82 ± 0.11 in the control group, and 87.49 ± 0.08 in the exposed group (Fig. 3C). This significant hypomethylation in miners’ leukocytes (*p* = 0.012), was dependent on one CpG site within the Alu sequence.

### 3.4 DNA methylation in antioxidant genes

**Fig. 4:**
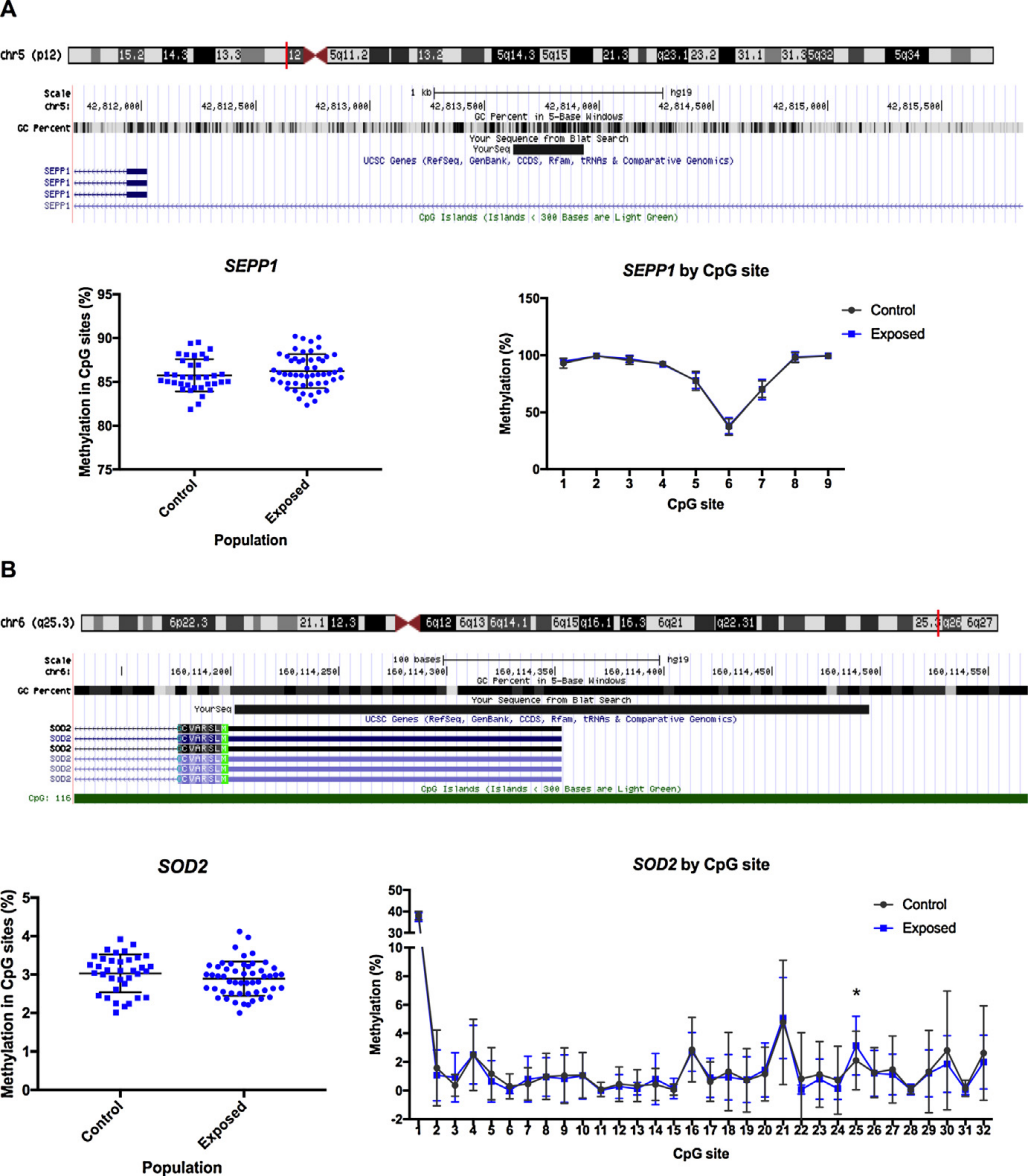
DNA methylation in antioxidant candidate genes. Methylation levels in the promoter of *SEPP1* (A) and *SOD2* (B) in peripheral blood samples of Hg exposed gold miners and control group. Genomic location targeted by specific primers in shown in the upper panel for each assay. Mean methylation for the targeted region is shown in the left panel, and methylation levels by CpG site is shown in the right panel. Unpaired t test, (*) p < 0.05.

In addition to global methylation, candidate genes linked to oxidative stress and DNA repair were selected for quantitative DNA methylation assessment. The *SEPP1* gene encodes for a selenoprotein that function as an antioxidant in the cell (Chen et al., 2006). Distribution of percentage methylation of *SEPP1* promoter is shown in Fig. 4. In average, the *SEPP1* methylation in the exposed individuals (86.23%) was higher than the controls (85.75%) but it was not significantly different. This same pattern was seen for the 9CpG sites evaluated (Fig. 4).

The *SOD2* gene codifies for an enzyme involved in the cell antioxidant response. On average, DNA methylation levels were not significantly different between the Hg exposed and controls (Fig. 4). Nevertheless, when analysing by individual CpG sites, a significant difference was found for site 25 (*p* = 0.022) showing hypermethylation in the exposed group with a difference of 1.04%.

### 3.5 DNA methylation in BER repair genes

**Fig. 5:**
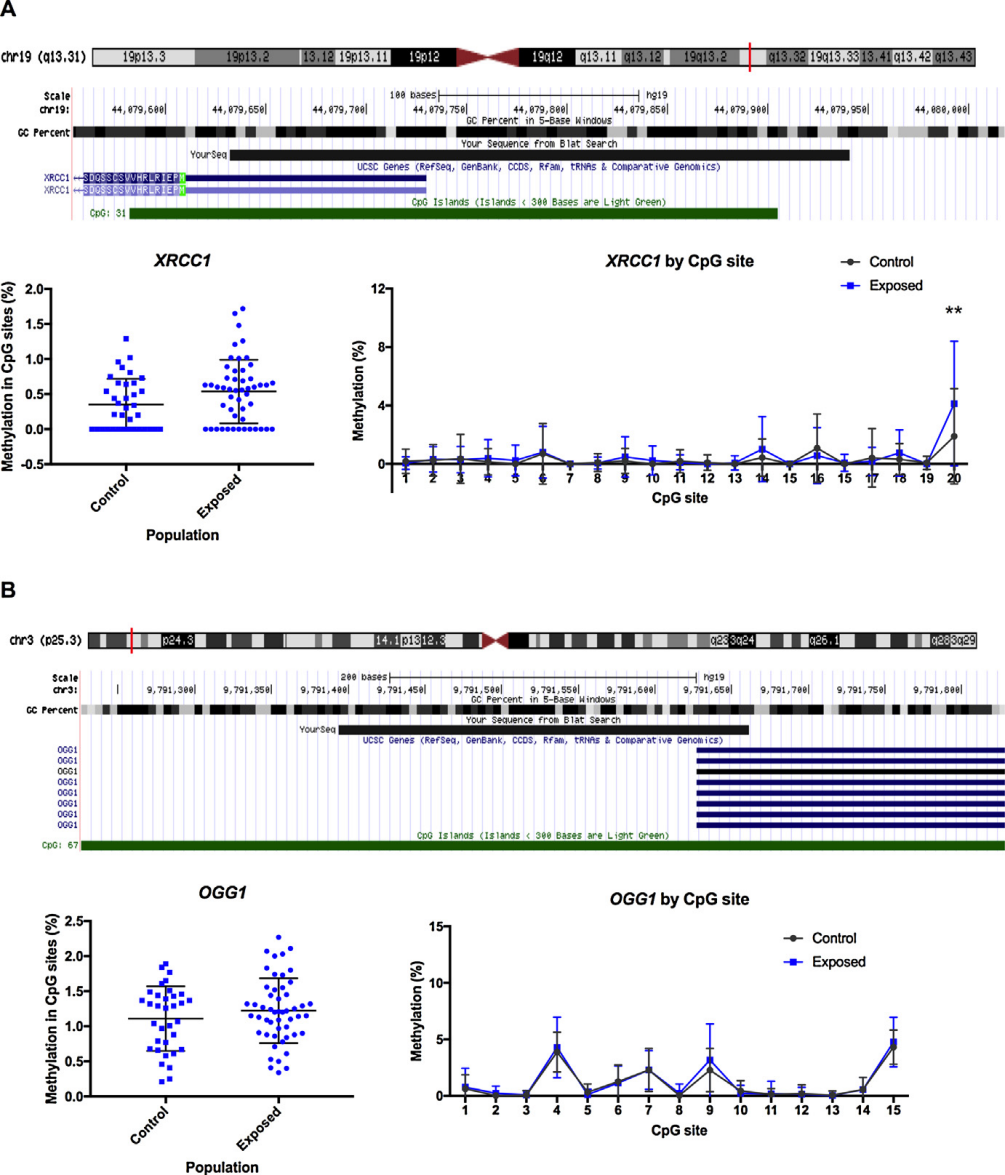
DNA methylation in DNA repair candidate genes. Methylation levels in the promoter of *XRCC1* (A) and *OGG1* (B) in peripheral blood samples of Hg exposed gold miners and control group. Genomic location targeted by specific primers in shown in the upper panel for each assay. Mean methylation for the targeted region is shown in the left panel, and methylation levels by CpG site is shown in the right panel. Mann-Witney U test, (**) p < 0.01.

As described above, oxidative stress may be induced by Hg occupational exposure; therefore, BER repair genes involved in the oxidative cell response were selected as candidate genes in this study. *XRCC1* and *OGG1* methylation was assessed in the study population. *XRCC1* methylation level is shown in Fig. 5. No significant differences were found for the average percentage of DNA methylation between the control (0.36%) and exposed subjects (0.52%), although the methylation level is increased in the miners. The percentage DNA methylation at individual CpG site showed a significant increase in site 20 (*p* = 0.007) for the exposed population with a difference of 2.24% (Fig. 5). In the case of the *OGG1* promoter, the average percentage of methylation was very similar for the controls (1.11%) and the miners (1.21%), as seen in Fig. 5. The same trend was found at the single-CpG site level.

### 3.6 DNA methylation and oxidative stress

**Fig. 6:**
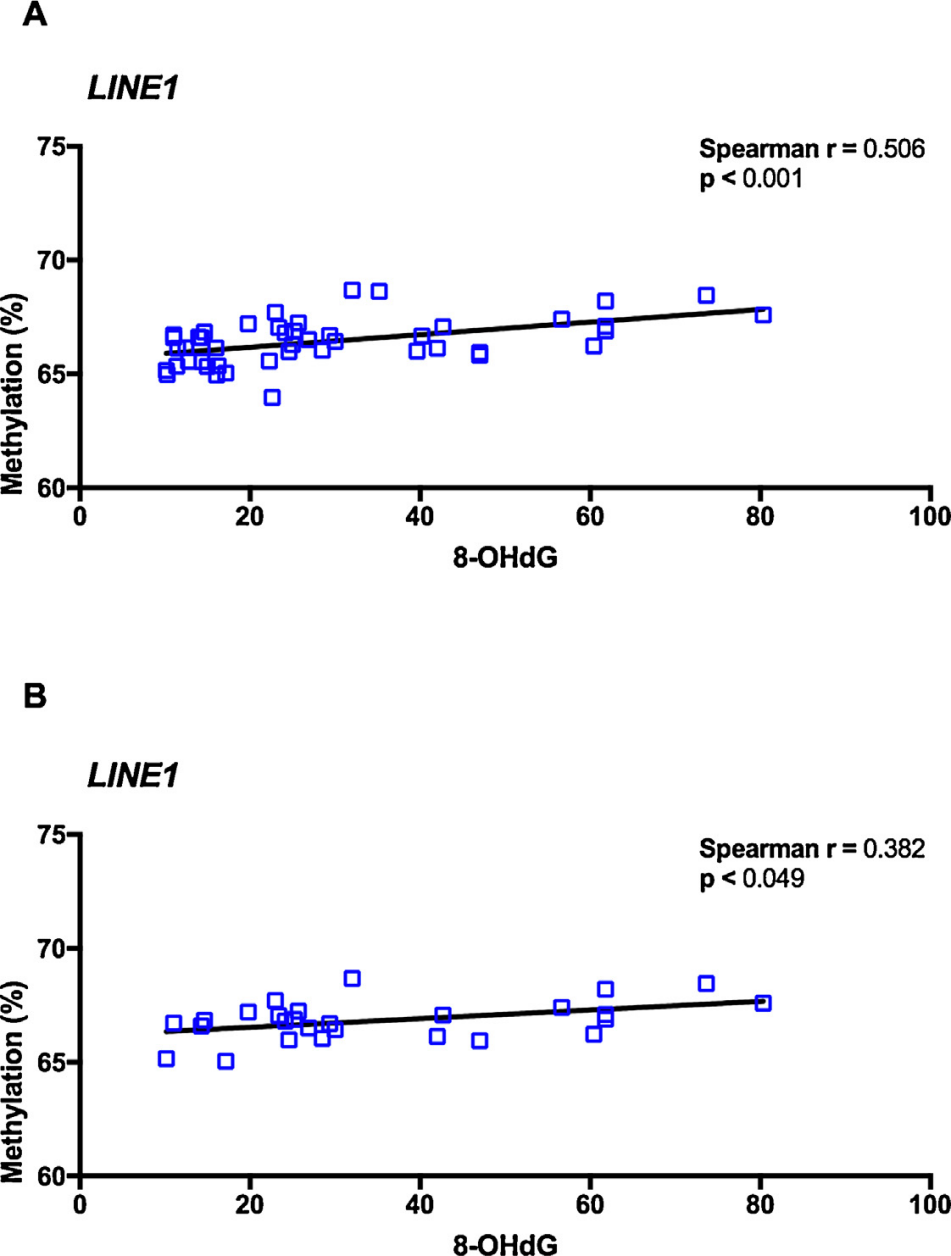
Correlation between global DNA methylation and oxidative stress. Bivariate correlations between *LINE1* methylation and oxidative DNA lesions in the general (A) and in the Hg occupationally exposed populations (B).

Bivariate correlations between methylation status and oxidative DNA lesions were evaluated in the total population (Fig. 6A). Significant positive correlation between 8-OHdG levels and *LINE1* methylation was found (Spearman r = 0.506, *p* < 0.001). Increasing values of 8-OHdG were associated with increasing percentage of *LINE1* methylation. The same relationship was observed when analyzing the exposed population separately (Spearman r = 0.382, *p* = 0.049) (Fig. 6B).

### 3.7 DNA methylation and Hg levels

**Fig. 7:**
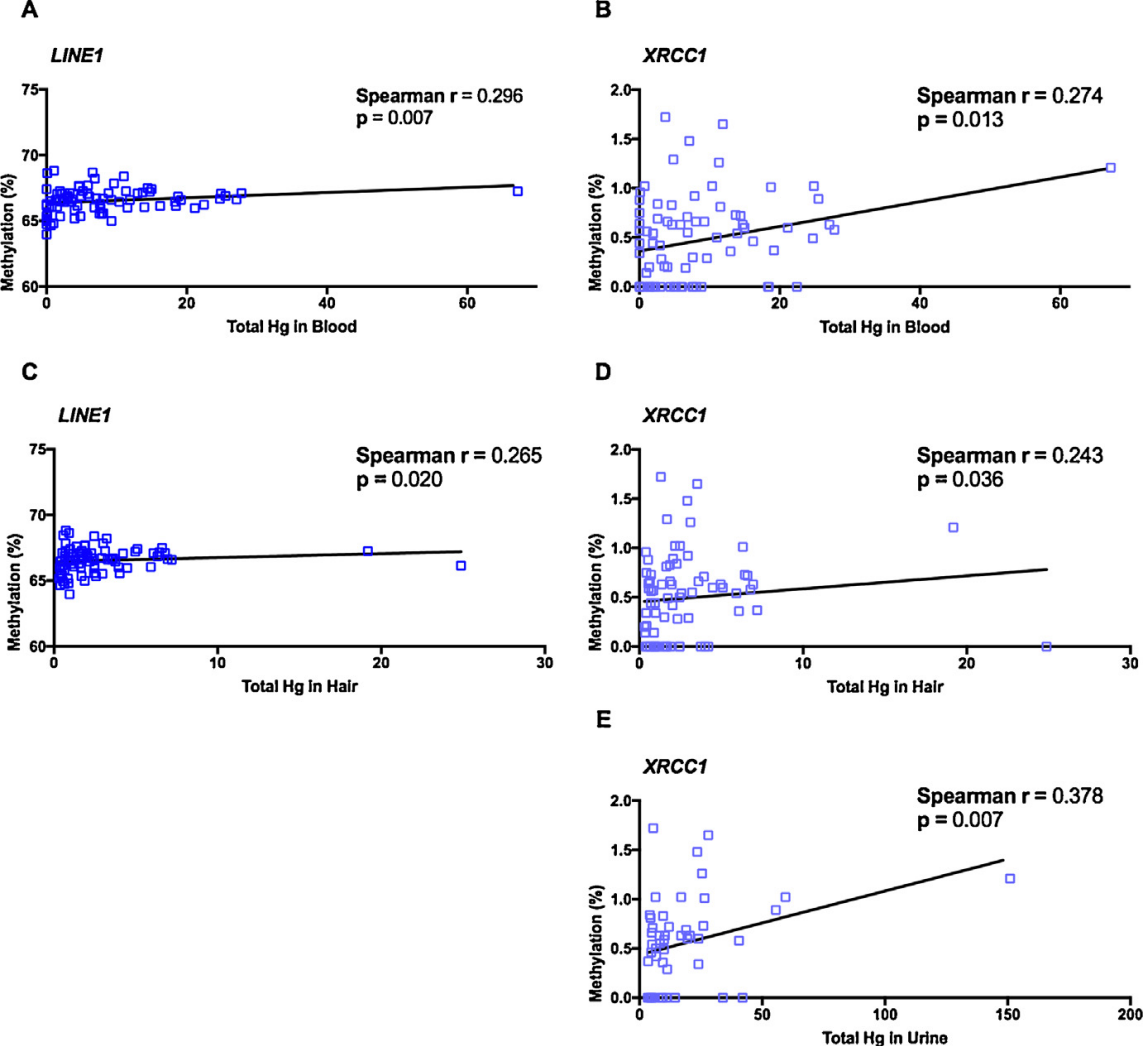
Correlation between DNA methylation and total Hg levels. Bivariate correlations between *LINE1* methylation and total Hg in blood (A) and hair (C) samples. Correlations between *XRCC1* methylation and total Hg in blood (B), hair (D) and urine (E) samples.

Bivariate correlations between methylation status and Hg levels were evaluated in the total population (Fig. 7). A positive correlation was found between blood Hg levels and *LINE1* and *XRCC1* methylation (Spearman r = 0.296, *p* = 0.007 and Spearman r = 0.274, *p* = 0.013, respectively). In the same way, hair Hg levels were positively correlated with *LINE1* and *XRCC1* methylation (Spearman r = 0.266, *p* = 0.020 and Spearman r = 0.243, *p* = 0.036, respectively). On the contrary, urine Hg levels were only correlated with *XRCC1* methylation (Spearman r = 0.378, *p* = 0.007).

## 4 Discussion

We have shown a higher Hg content in different tissues from artisanal gold miners when compared to control subjects. Methylation changes in *Alu* and *LINE1* repetitive elements and *SOD2* and *XRCC1* promoters were found in miners’ blood. Furthermore, our data revealed oxidative stress as DNA oxidative lesions in the miners. We found that higher levels of oxidative stress, were correlated with *LINE1* hypermethylation, and *LINE1* as well as *XRCC1* methylation were positively correlated with Hg exposure.

The main source of Hg exposure in the artisanal gold mining process is the inhalation of elemental Hg (Hg°) vapour. Biomarkers of Hg exposure focus on detecting total Hg levels in biological samples. Hg concentration in urine is the best biomarker of chronic exposure to Hg° and inorganic Hg, as urinary Hg levels derives directly from deposits in the kidney tissue (Park and Zheng, 2012). We showed higher levels of total Hg in urine in the miners. On the other hand, in the general population total Hg in blood is used as a measure of MeHg, which is associated with fish consumption, with the assumption that inorganic Hg exposure is low (Berglund et al., 2005). Therefore, the exposure to MeHg will depend on fish intake and the concentration of metal in the fish (de Oliveira et al., 2014). In this study, we found that the non-occupational exposed population report a significant higher frequency of fish consumption (2–4 times per week) than the miners (just once a month). It has been demonstrated that after contaminated fish ingestion, a small Hg fraction of only 5% may be in the blood (Clarkson et al., 2007). Our results showed higher levels of total Hg in blood from the miners when compared to the controls, which can be associated not only with MeHg, but with Hg° vapour exposure due to the low frequency of fish consumption in this population. It is important to take into account that blood Hg level, as a measure of inorganic Hg, decreases within days of exposure, and do not correspond to total body burden (Park and Zheng, 2012), since inorganic Hg binds weakly to red blood cells and Hg found in plasma corresponds to glomerular filtration (Clarkson et al., 2007). In the same way, total Hg levels in hair are used as a measure of MeHg long term exposure (Berglund et al., 2005). MeHg exposure due to fish consumption mainly accords to total Hg in hair (Díez et al., 2008). As mentioned before, we found that the control group consumed more fish than the occupationally exposed population; however, total Hg levels in hair from this group were lower. One study on a similar population revealed that there were not differences in hair total Hg concentrations when comparing different frequencies of fish consumption due to the main consumption of non-carnivorous fish species with low Hg concentrations (Olivero-Verbel et al., 2011), which may be explaining the low Hg levels found in hair from the control group in our study. Even though hair Hg is not recommended for biological monitoring of Hg° exposure, significant correlation between hair total Hg and urine Hg in artisanal mining workers suggest that hair can be a biomarker of Hg vapour exposure (Li et al., 2011). Again, hair Hg levels were high within the miners in this study and significantly correlated with urine levels, thus occupational Hg exposure was confirmed in the three samples analysed.

Exposure to Hg is a major risk for human health, with Hg toxicity appearing even at low levels of exposure (Lee et al., 2012). Such toxic effects of Hg are principally due to increased ROS production, cellular lipid peroxidation, and inhibition of antioxidant enzymes (Crespo-López et al., 2009; Ercal et al., 2001). Hg, like other metals, produces the superoxide anion radical (O·^2^) and hydroxyl radical (·OH) via Fenton reaction (Koedrith and Seo, 2011; Lee et al., 2012). In line with this, our results suggest the induction of oxidative stress by occupational Hg exposure, reflected in high levels of 8-OHdG in gold miners. The 8-OHdG is the most frequent modified base produced by oxidative DNA damage. Of note, DNA damage in cells chronically exposed to oxidative stress results in DNA breaks, base modifications, DNA mutations, genomic instability (Dizdaroglu, 2012), and eventually carcinogenicity (Lee et al., 2012).

Oxidative stress as primary mechanism involved in metal-toxicity causes genetic and epigenetic changes. One commonly reported alteration is global hypomethylation (Hou et al., 2012). In general, global hypomethylation has been associated with cancer development by inducing chromosomal instability and alteration in transcription of otherwise silenced adjacent genes (Herceg and Vaissière, 2011; Hou et al., 2012). We found a significant decrease in DNA methylation of *Alu(Yb8)* in peripheral blood from gold miners. Alu elements are short interspersed elements (SINEs) that are part of the primate specific retrotransposons, with the Yb- lineage being one of the young group of evolutionarily-related *Alu* subfamilies in humans (Carter et al., 2004). Due to its high density of CpG, Alu elements may be more affected in terms of DNA methylation (Xing et al., 2004), and *Alu(Yb8)* hypomethylation has been associated with tumorigenic events suggesting that these elements may be used as biomarkers for early cancer detection (Bakshi et al., 2016). In contrast, and contrary to expectations, we found that *LINE1* methylation was significantly higher among the miners, and it was correlated with 8-OHdG and Hg levels. LINE1 elements are found in 20% of mammalian genome approximately (Isabelle Miousse et al., 2015), and are AT-rich genomic regions (Akers et al., 2014). *LINE1* hypermethylation was found in mice after exposure to ionizing radiation (Koturbash et al., 2016; Prior et al., 2016), in this case changes in DNA methylation were associated with time after exposure and the evolutionary age of the transponsable element. Alterations in DNA methylation associated with environmental stressors are not unidirectional and may vary by loci (I R Miousse et al., 2014). Given the nature of Alu and LINE1 elements, methylation may be differentially regulated (Akers et al., 2014). Additionally, it has been shown that different transponsable elements could be differentially methylated in response to exposure to the same metal within the same experimental system (Isabelle R. Miousse et al., 2015). Nevertheless, the different methylation changes observed in our study could be a result of blood cell composition, as differences in DNA methylation have been reported in different blood cell types (Wu et al., 2011).

Hg exposure modulates gene expression in biological pathways such as stress response and DNA repair (Koedrith et al., 2013) that may be explained by epigenetic mechanisms. We analysed DNA methylation changes in antioxidant response (*SEPP1* and *SOD2)* and BER repair pathway (*XRCC1* and *OGG1*), and found no significant differences between the gold miners and control groups. *SEPP1* promoter hypomethylation was found in male dental professionals associated with MeHg exposure with a general mean of 37% (Goodrich et al., 2013); our results indicate a mean of *SEPP1* promoter methylation around 86%, which suggest that methylation pattern in this gene may be influenced by the different kind of Hg exposure. Even though we did not find significant differences between the two populations for *SOD2* methylation, when analysing individual CpG sites we found significant hypermethylation only in one site. *SOD*2 hypermethylation induces SOD2 downregulation impairing redox signalling and creating a proliferative, apoptosis-resistant state as the one seeing in carcinogenesis (Archer et al., 2010). In the same way, differential *XRCC1* hypermethylation was only found in one CpG site for the gold miners. But hypermethylation for this gene was correlated with high levels of Hg both in hair, blood and urine suggesting an effect of exposure in the methylation pattern. The results of differential methylation on isolated CpG sites should be taken with care, as methylation status within a given promoter tends to change in a coordinated manner for most cytocines.

In addition to blood cell mixture, further limitations of our study include the lack of a control group of subjects not exposed to Hg, as our control individuals were exposed to low levels because they were habitants of the same location of the miners. Although methylation changes found in this study were statistically significant between gold miners and controls, the size effects were small and the methylation levels often overlap between the two populations suggesting that further studies will be necessary to identifying suitable epigenetic biomarkers for risk assessment of occupational Hg exposure. Knowledge generated from this kind of research may contribute to justifying environmental policies for reducing the impact of this exposure on miners’ health.

## Declarations

### Author contribution statement

Diana M. Narvaez: Performed the experiments; Analyzed and interpreted the data; Wrote the paper.

Helena Groot: Conceived and designed the experiments.

Sonia M. Diaz; Ruth M. Palma: Conceived and designed the experiments; Contributed reagents, materials, analysis tools or data.

Nathalia Munoz, Marie-Pierre Cros: Contributed reagents, materials, analysis tools or data.

Hector Hernandez-Vargas: Conceived and designed the experiments; Analyzed and interpreted the data; Wrote the paper.

### Funding statement

This work was supported by INS as part of the surveillance program, Human Genetics Laboratory of Universidad de los Andes, and Inserm (Institut National de la Santé et de la Recherche Médicale) Plan Cancer 2014-2019, Numéro de Projet: C14088CS.

### Competing interest statement

The authors declare no conflict of interest.

### Additional information

No additional information is available for this paper.
